# The role of nicotinic cholinergic neurotransmission in delusional thinking

**DOI:** 10.1038/s41537-020-0105-9

**Published:** 2020-06-12

**Authors:** Michael Caton, Enrique L. M. Ochoa, Francisco J. Barrantes

**Affiliations:** 1 The Permanente Medical Group, Kaiser Santa Rosa Department of Psychiatry, 2235 Mercury Way, Santa Rosa, CA 95047 USA; 2 Heritage Oaks Hospital, 4250 Auburn Boulevard, Sacramento, CA 95841 USA; 3 Volunteer Clinical Faculty, Department of Psychiatry and Behavioral Sciences, University of California at Davis, 2230 Stockton Boulevard, Sacramento, CA 95817 USA; 4 Laboratory of Molecular Neurobiology, Institute for Biomedical Research (BIOMED), Faculty of Medical Sciences, UCA-CONICET, Av. Alicia Moreau de Justo 1600, C1107AFF Buenos Aires, Argentina

**Keywords:** Schizophrenia, Psychosis

## Abstract

Delusions are a difficult-to-treat and intellectually fascinating aspect of many psychiatric illnesses. Although scientific progress on this complex topic has been challenging, some recent advances focus on dysfunction in neural circuits, specifically in those involving dopaminergic and glutamatergic neurotransmission. Here we review the role of cholinergic neurotransmission in delusions, with a focus on nicotinic receptors, which are known to play a part in some illnesses where these symptoms appear, including delirium, schizophrenia spectrum disorders, bipolar disorder, Parkinson, Huntington, and Alzheimer diseases. Beginning with what we know about the emergence of delusions in these illnesses, we advance a hypothesis of cholinergic disturbance in the dorsal striatum where nicotinic receptors are operative. Striosomes are proposed to play a central role in the formation of delusions. This hypothesis is consistent with our current knowledge about the mechanism of action of cholinergic drugs and with our abstract models of basic cognitive mechanisms at the molecular and circuit levels. We conclude by pointing out the need for further research both at the clinical and translational levels.

## Introduction

DSM-5^1,2^ defines delusions as fixed beliefs that are not amenable to change in light of conflicting evidence. Of note, the new edition of DSM captured an important nuance, namely that it is the dysfunctional process of belief, rather than its content necessarily being false, which is significant. This definition highlights the central differentiating aspects of delusions, which allow us to identify them in the clinic and distinguish them from other kinds of aberrant beliefs, including non-pathological ones: in particular, the fixedness of the belief (conviction) and cultural non-appropriateness (idiosyncrasy). Another important aspect of delusional belief is its specificity: delusions tend to cluster into certain themes, such as persecutory, grandiose, erotomanic, or somatic. That these themes are constant across cultures suggests an underlying biology. Furthermore, it has long been recognized that there is a difference between somatic delusions—e.g. Capgras, Fregoli, reduplication, or arguably, neglect syndromes, in which the patient is often remarkably disinterested in connecting to other beliefs and experiences—and the themed delusions named previously.

Both hallucinations and delusions are present in many neuropsychiatric illnesses. The classical definition of delusions as false beliefs, and hallucinations as perceptions without corresponding external stimuli, have been criticized as oversimplistic^3^. Delusions only rarely occur in isolation from other psychotic symptoms^4^, although this does occur in the so-called delusional disorder^2,5^. Typically, research on delusions has focused on the wide palette of schizophrenia spectrum disorders, but the basic mechanisms of delusion formation are shared among multiple disease states^6^. In particular, delusions occur in Alzheimer, Parkinson, and Huntington diseases (HDs), delirium, post-ictal states and bipolar disorder among others, different illnesses presenting with a differential predominance of delusional themes and duration of symptoms, and sometimes correlating with other symptoms such as perceptual disturbances^7^.

Dysfunction of the dopaminergic system has traditionally been assigned the central role in the pathogenesis of psychotic symptoms^8^. In health, dopamine (DA) neurotransmission mediates the motivational salience of environmental rewards. In psychosis, salience appears not as a result of exogenous stimuli but rather stems from “an endogenously driven assignment of novelty and salience to stimuli”^8,9^. Anatomically, aberrant salience is mediated by midbrain dopaminergic projections to mesocorticolimbic areas^8,9^. It is for this reason that among the illnesses discussed here, dementia in AD is particularly interesting, because it is primarily a cholinergic disturbance^10^.

Somatic delusions frequently occur in psychotic patients where they accrete more polythematic-like explanations, but they can also occur outside a network of other delusional beliefs, remarkably like the syndromes observed in neurological patients with imageable lesions in their right frontal lobe^11^. The two-factor hypothesis of delusions^12–16^ accounts for these differences and puts forward a framework that synthesizes imaging work and recent models of delusional thinking, focusing on right frontal hypofunction and dorsal striatal hyperfunction, and accounting for specific features of delusions.

The effects of antipsychotics on the reasoning bias (“jumping to conclusions”, reduced belief flexibility, an externalized attributional style, and deficits in “theory of mind”) are well known. Antipsychotics improve “theory of mind” and mainly belief flexibility, with little effect on the “jumping to conclusions” trait^17^. This review will not focus on the therapeutics of delusions which include medications and special types of psychotherapy, for which the reader is referred to an excellent review on the subject^18^.

## The molecular and circuit levels

### Functional roles of the cholinergic system in mammalian cognitive processes

Neuronal nicotinic acetylcholine receptors (nAChRs) are a heterogeneous family within the superfamily of pentameric ligand-gated ion channels (pLGIC) that are expressed throughout the brain and involved in a wide range of physiological and physiopathological processes^19–24^. pLGIC include not only the neuronal subtype but also the muscle-type nAChRs, γ-amino butyric acid (GABA)_A_, glycine, and the 5-HT3 subtype of serotonin receptors.

Although brain nAChRs share a common structural architecture, their cerebral localization, pharmacology, biophysical properties, and developmental occurrence differ. The homomeric (α7 and α9) or heteromeric (α2–α7 combined with β2–β4) nAChR subtypes result from the combination of these subunits into pentameric oligomers of ca. 270,000 Da (see reviews in refs ^24,25^). Through this combinatorial procedure, Nature has developed abundant diversity to fulfill connectomically and topologically distinct functional requirements. Brain nAChRs have been classically associated with excitatory neurotransmission^26^ and its modulation (see ref. ^27^), but these are only fragmentary facets of their functional palette; their cellular distribution along development and adulthood^28,29^ and subcellular localization (predominantly presynaptic, but also perisynaptic and postsynaptic^30^; reviewed in ref. ^24^) adds complexity to this apparently primary role. Thus, nAChRs can modulate the release of other neurotransmitters^31,32^, or acetylcholine (ACh) itself^33–35^, regulate synapse development^36^, cell viability^37^, neurite outgrowth^38^ and brain energetic homeostasis^39^, and modulate the establishment of functional networks during neural development^40^ (see review in ref. ^41^), apparently affecting the neuronal survival/death homeostasis. Because of their strategic location in areas involved in memory acquisition, maintenance and retrieval, nAChRs in hippocampus and cerebral cortex play major roles in cognition and mnemonic functions^42,43^ and modulate prefrontal cortex functions related to conscious processing^44^. All these phenomena are subject to dysfunction under disease conditions (see below). At present, we probably know only a minute fraction of the roles played by native nAChR subtypes in specific functions in the human brain.

The most widely expressed subtypes in brain are the heteromeric α4β2-subtype and the homomeric α7 nAChRs^24^. These receptors are essentially ionotropic proteins, i.e. ion channels, whose main ability is to rapidly permeate potassium, sodium, and calcium ions in neurons and astrocytes. It has recently been disclosed that α7 nAChRs have an additional property^45,46^, namely the ability to activate downstream metabolic cascades much like the G-protein-coupled receptors, which constitute a structurally and evolutionarily unrelated superfamily of receptors. This dual functionality only found so far in the α7 nAChR, couples the ionotropic activation elicited by the endogenous agonist ACh to metabotropic neuronal signaling and plasticity phenomena mediated by a G-protein-binding domain located in the intracellular loop of the α7 nAChR. Thus, neuronal α7 nAChRs appear to display both ionotropic and metabotropic properties, whereas α7 nAChR in microglia lack ion channel permeation and exhibit only the metabotropic function, as is the case with other non-neuronal cells, like macrophages, which operate via the JAK2/STAT3 transcription factor pathway, as reviewed elsewhere^24^.

The α7 nAChR, encoded by the *CHRNA7* gene^47^, has been the prototype of the homomeric (five apparently identical copies, α7 × 5) type of neuronal nAChRs. Another peculiarity of the α7 nAChR is that it possesses five identical ACh recognition sites instead of the canonical two sites of the other nAChRs: heteromeric subtypes carry the two orthosteric agonist-recognition sites at the interfaces between two adjacent non-homologous subunits, one of which is an α-subunit. Gotti and coworkers have characterized a native heteromeric α7β2 nAChR expressed in human basal forebrain and having physiological and pharmacological profiles different from those of the typical homomeric α7 nAChR^48^ reviewed in refs. ^24,49^. The latter subtype of neuronal nAChR is highly expressed in brain, particularly in cerebral cortex, subcortical areas, and hippocampus, where it has been associated with mechanisms of neuroprotection and the processes of learning, memory, attention, reward, sensory information processing, and cognition^50,51^.

Recent experimental work has provided new information on the distribution of nAChRs across the different layers of the cerebral cortex and the different functional roles played by these layer-specific receptors, either located on dendrites of principal neurons or on GABAergic interneurons^52^. An interesting finding is that the spike timing-dependent synaptic plasticity is oppositely regulated by the activation of nAChRs located in different cortical layers: superficial layer 2/3 (L2/3) pyramidal neurons are inhibited by nAChR activation on interneurons, whereas deep L6 pyramidal neurons are excited by postsynaptic nAChRs. Thus, this stratified nAChR expression allows functional layer-specific control of cortical processing and plasticity by the basal forebrain cholinergic neurons. This system appears to be evolutionarily conserved from mice to humans, the neocortex of the latter maintaining opposite layer-specific cholinergic control of synaptic plasticity. Different sets of cholinergic neurons located in the basal forebrain preferentially target superficial or deep cortical layers of the medial prefrontal cortex (mPFC)^52^.

Abundant layer-specific neuroanatomical representation of these projections in brain are an important manifestation of phylogenetic evolution; however, it is the diversity and extent of expression of nAChR subtypes at the cellular and subcellular levels, and the multiple combinations of subunits in this pentameric receptor, that play a major role in the physiology and pathology of the nicotinic cholinergic system in brain. The correlation between layer-specific nAChR expression and functional layer-specific control of cortical processing and plasticity by the basal forebrain cholinergic neurons^52^ is a clear example of the strategy developed by the brain in the course of evolution to maximize structural–functional diversity at the cellular level. The nine α subunits and three β subunits so far identified in the CNS already provide combinatorial potential, which to date, however, appears not to be fully realized in the actual brain. This repertoire of combinatorial possibilities also determines various levels of affinity for the neurotransmitter and cholinomimetic drugs as well as the topographical specialization of nAChRs across the brain architecture, the ion permeability, and various degrees of desensitization resulting from ligand binding to different nAChR subtypes. For instance, the α7 nAChR has an unusually fast on-rate of desensitization, which apparently plays a functional role in the termination of synaptic transmission mediated by this subtype of receptors. The natural ligand, ACh, and the full agonist nicotine trigger sodium flux through the α4β2 nAChR and calcium-mediated currents in the case of the homo-pentameric α7 nAChR, albeit with 100–1000 lower affinity in the latter case^53^.

Abnormal expression of α4β2 nAChRs has been shown to alter cholinergic neurotransmission in neuropsychiatric disorders, including autism spectrum disorders^54,55^, nicotine addiction^56–60^, AD^61^, and Parkinson disease (PD)^56^. Alterations of α7 nAChR expression have been associated with various brain disorders, interactions with amyloid-β, and with the pathogenesis of dementia through multiple mechanisms^62–69^. Expression of the α7 nAChR, which binds nicotine with low affinity, is reduced in the hippocampus of schizophrenic patients^70^. Early studies found a non-uniform decline in α7 nAChR expression; regional specificity was suggested by the diminution of expression in frontal cortex but not in parietal cortex^71^.

The experimental evidence accumulated during the last two decades provides solid support to the notion that α7 nAChR is a strong candidate for understanding the pathophysiology of psychosis in both schizophrenia spectrum disorders and AD^72,73^. Abnormal expression of α7 nAChR has also been associated with the auditory sensory gating deficit characteristically found in patients suffering some forms of schizophrenia spectrum disorders and their relatives. The typical sign is the diminished suppression of an auditory-evoked response (P50) to repeated stimuli. The *CHRNA7* gene coding for the α7 nAChR protein has been linked to the P50 deficit^74^. This finding, in conjunction with evidence of familial transmission of this sensory gating deficit, led to a suggested pathogenic role of the gene coding for the α7 nAChR in schizophrenia spectrum disorders^75^ (see also refs. ^76–79^ for recent reviews of genetic factors in these disorders). Hashimoto^80^ has prompted the use of auditory sensory gating as a translational biomarker.

Additional associations are found between nicotine addiction and risk of dementia. The comorbidity of nicotine dependence is 2–3-fold higher in psychiatric patients in general, but the association between tobacco smoking and schizophrenia is even stronger, as shown in meta-analysis of worldwide studies: 70% of male schizophrenic patients and 40% female patients were found to be smokers^81^, a prevalence higher than for any other psychiatric disease. Another linkage between the smoking habit and illnesses is that smoking promotes atherosclerosis and vascular disease increases the risk of dementia, whereas nicotine has been shown to exert neuroprotective effects on dementia via α7 nAChRs in preclinical studies^82^. Antagonists and agonists of α7 nAChRs have been found to regulate the gating or filtering of auditory information in both humans and animal models^83–87^.

[H^3^]-nicotine binding, an in vitro biochemical readout of the high affinity nAChRs, was also found to be lower in patients with schizophrenia spectrum disorders and did not increase in response to tobacco use, as observed in control subjects^65^. It is hypothesized that patients smoke to alleviate cognitive symptoms^57^. This is taken as evidence that nicotine enhances cognition^88^. The hypothesis is still inconclusive; see recent review in ref. ^89^.

A recent study focuses on the relationship at the gene–gene and gene–environment levels of the interactions between *CHRNA7* polymorphism, apolipoprotein E (*APOE*) ε4 carriers, and smoking on risk of dementia. Late-onset AD (LOAD) patients and 115 vascular dementia patients were compared with healthy individuals. Among *APOE*ε4 non-carriers*, CHRNA7* rs7179008 and GT haplotype in *CHRNA7* block4 variant carriers exhibited significantly decreased risks. These findings suggest that there are gene associations that up-regulate or down-regulate the risk of vascular and non-vascular forms of dementia^90^.

## Role of muscarinic receptors in psychotic disorders

Although this review addresses primarily the role of nicotinic cholinergic neurotransmission in delusional thinking, we will briefly review the brain muscarinic system and the evidence that points towards its involvement in psychotic states^91–94^.

Central muscarinic receptors are G-protein-coupled receptors widely distributed in the mammalian brain. They consist of five subtypes termed M1–M5 and called M1-like and M2-like receptors. M1-like receptors comprise the M1, M3, and M5 receptors. These are located post-synaptically, promoting neurotransmission when activated by ACh. M2-like receptors comprise the M2 and M4 receptors and are located both pre-synaptically and post-synaptically, acting mainly as autoreceptors (for detailed reviews of the central muscarinic system see refs ^95,96^.

The M1 receptor is the most abundant muscarinic receptor in the cortex, the striatum, and the hippocampus. The M3 receptor shows a distribution which parallels that of the M1 receptor, albeit with a lower density. The nucleus basalis of Meynert and the occipital cortex are rich in M2 receptors. M4 receptors are prominent within the striatum and the caudoputamen. The M5 is the muscarinic receptor with the lowest expression levels; it is found in the substantia nigra pars compacta and the ventral tegmental area^97–99^.

The clinical entity known as anticholinergic delirium observed in patients treated with anticholinergic drugs suggests that muscarinic receptors are implied in these psychotic phenomena^100–102^.

In 1972, Janowsky and coworkers advanced a cholinergic–adrenergic hypothesis of mania and depression^103^. The authors proposed a balance between cholinergic and adrenergic neurotransmission in those areas of the brain involved in affective regulation. They briefly mention the exacerbation of schizophrenic symptoms in patients exposed to organophosphates^104^, suggesting a muscarinic cholinergic receptor mechanism after cholinesterase inhibition. Due to increased ACh at the synaptic cleft in multiple brain areas after organophosphate exposure, it is difficult to discriminate between muscarinic and nicotinic receptors in the production of this exacerbated symptomatology.

A more direct involvement of muscarinic receptors in the production of psychosis (in this case negative symptoms of schizophrenia) was advanced by Tandon and Greden^105^. Positive symptoms of schizophrenia are explained by increased mesocortical and mesolimbic dopamine but a compensatory increase in cholinergic mechanisms is operative in the production of negative symptoms such as alogia, avolition/apathy, anhedonia, and attentional deficits^105^. This involvement of cholinergic mechanisms and more specifically of muscarinic receptors is supported by a study that shows amelioration by the anticholinergic agent trihexiphenidyl of negative symptoms in schizophrenia patients. Trihexiphenidyl binds to the M1 muscarinic receptor^106^. This may partially explain the elevation in mood, better socialization, and stimulation effected by trihexyphenidyl in this patient population, often subsequently leading to abuse of this medication^107^. The effect is also observed in neuroleptic-naïve patients indicating a beneficial effect beyond relief of extrapyramidal side effects^105^.

The evidence for involvement of muscarinic receptors in schizophrenia spectrum disorders is supported by post-mortem, pharmacological, and imaging studies^94^. In this respect, an important study by Bakker and collaborators^108^ was able to show a correlation between a reduced binding potential of M1 receptor in the dorsolateral prefrontal cortex, a key area involved in the production of negative symptoms in schizophrenia spectrum disorders. The study was conducted on medication-naïve psychotic patients and used single-photon emission computed tomography with a specific radioligand that measured binding potential of M1 receptors^108^. The authors were able to show a correlation between lower binding potential of the M1 receptor and poor outcomes in several neurocognitive tests and negative symptoms severity. However, there was no mention of delusional thinking.

In conclusion: it is clear from the published evidence regarding the involvement of muscarinic receptors in psychosis, specifically in schizophrenia spectrum disorders, that these receptors are related to cognitive deficits in attention, working memory, concentration, and executive functions^91,93,108^, which in turn translate into negative symptoms, and in neurological soft signs^57^. Although short-lived delusional thinking is certainly a feature of anticholinergic delirium produced by antimuscarinic drugs^101,102^, it is not clear how muscarinic receptors participate in the genesis and ulterior maintenance of both well-crystallized and systematized delusional thinking.

## Cholinergic pathways in the brain

The cholinergic system consists of subcortical as well as cortical domains, organized into two main pathways: (i) the brainstem and (ii) the magnocellular basal forebrain cholinergic system^109^.

There are two cholinergic nuclei in the brain, as well as a high concentration of cholinergic interneurons in the basal ganglia. Interactions between cholinergic interneurons and dopaminergic neurons in the basal ganglia are well characterized. The striatum contains extremely high concentrations of ACh relative to other areas in brain, the main locus being in the cholinergic interneurons, specifically in the dorsal striatum (caudate nucleus and putamen), which however constitute only 1–2% of all the striatal cellular content. The mesocorticolimbic system connects the neurons of the ventral tegmental area (VTA) with the nucleus accumbens (NAc, in the ventral striatum) and the prefrontal cortex (PFC). The experience-dependent dopamine neurons in the VTA are key elements in the regulation of the brain reward system, whose dysregulation is associated with depression and mood disorders^110,111^. Excitatory glutamatergic/cholinergic inputs in dopaminergic neurons, as well as inhibitory GABAergic afferents on these cells, are modulated by nAChRs present in cholinergic, glutamatergic, and GABAergic terminals. A direct activation of excitatory postsynaptic nAChRs results in depolarization and firing of dopaminergic neurons^111^.

In addition, an indirect mechanism involving α7 nAChRs triggers glutamate release in glutamatergic synapses, which in turn elicits activity of dopaminergic neurons in the VTA^112^.

## ACh’s role in delusions in Alzheimer disease (AD)

Forty-one percent of persons with AD experience hallucinations and delusions early in the course of the disorder^113^. The occurrence of these symptoms is associated with faster cognitive decline^114^. Of particular relevance to this review, psychotic thinking consists mainly of persecutory and misidentification delusions^115^. Cholinergic deficits are implicated in the genesis of cognitive and psychotic symptoms in AD^116^, and these deficits have been associated with α7 nAChRs in both schizophrenia spectrum disorders and AD^72–75,84,85,117,118^. Furthermore, the gene coding for the α7 nAChR (*CHRNA7*) is known to be associated with symptoms of schizophrenia spectrum disorders in linkage and association studies^47,119^. More specifically, a strong correlation between delusional thinking in AD and a C/T polymorphism located in intron 3 of *CHRNA7* was reported by Carson and coworkers^72^. Delusional symptoms (mainly of the persecutory and misidentification types) were more pronounced in patients homozygous for the T allele than for the CC or the CT genotypes^72^.

Cholinergic brain pathways are implicated in cognitive processes^57,120^. The best example is the cognitive decline seen in patients with AD dementia, which is correlated with early degeneration of ACh-synthesizing neurons in the subcortical nuclei of the human basal forebrain^121–123^. New evidence suggests that the cholinergic system may be implicated not only in cognitive deficits but also in the development of psychotic symptoms in this patient population^10,101,124^.

A biochemical study examining regions of interest in post-mortem brains of patients with Lewy body dementia found an elevated M1-subtype of muscarinic ACh receptor concentration in the fusiform gyrus of the temporal lobe^125^; this type of receptors has not been found on dopaminergic neurons in the striatum^110,123^. In relation to this observation, it is instructive that the relationship between muscarinic tone and psychosis is reversed in schizophrenia spectrum disorders, where decreasing muscarinic tone is associated with increasing psychosis^126^ and agonism with decreasing psychosis^127^. Although the exact mechanism of action by which cholinergic deficits produce psychosis in AD is not known, imaging studies show that psychosis is associated with abnormalities in frontal and temporal cortices where cholinergic deficits are more pronounced^124^. The same brain areas show increased M2-type muscarinic receptor binding^128^, suggesting participation of these receptors in psychosis. It has been suggested that deficits in pathways involving muscarinic receptors abnormally facilitate dopaminergic systems producing psychosis^129^. Blockade of cholinergic circuits can produce psychosis, as seen in patients treated with anticholinergic drugs^102^. Anticholinergic drug-induced delirium, another example of psychosis seen in clinical settings, manifests with frank delusional thinking^124^. Drugs that enhance cholinergic transmission, such as cholinesterase inhibitors, decrease delusions and hallucinations in AD patients^130,131^.

## ACh in schizophrenia spectrum disorders

The relationship between nicotinic signaling and schizophrenia is complex, given the number of subunit combinations of this neurotransmitter receptor possibly involved and their distribution within the central nervous system. However, although the involvement of nicotinic signaling in the general cognitive decline in schizophrenia spectrum disorders is clear^57,120,132^, as is its potential to modestly improve cognition, this alone is insufficient to suggest participation of the nicotinic type of neurotransmission in delusions, as there is no consensus on the relationship between cognitive deficits of schizophrenia and the genesis and maintenance of delusional thinking^133,134^. It is also clear that a4β2 receptors are specifically upregulated in the striatum in smokers in general but not at all or at least not to the same degree in smokers with schizophrenia spectrum disorders^135,136^.

**Fig. 1:**
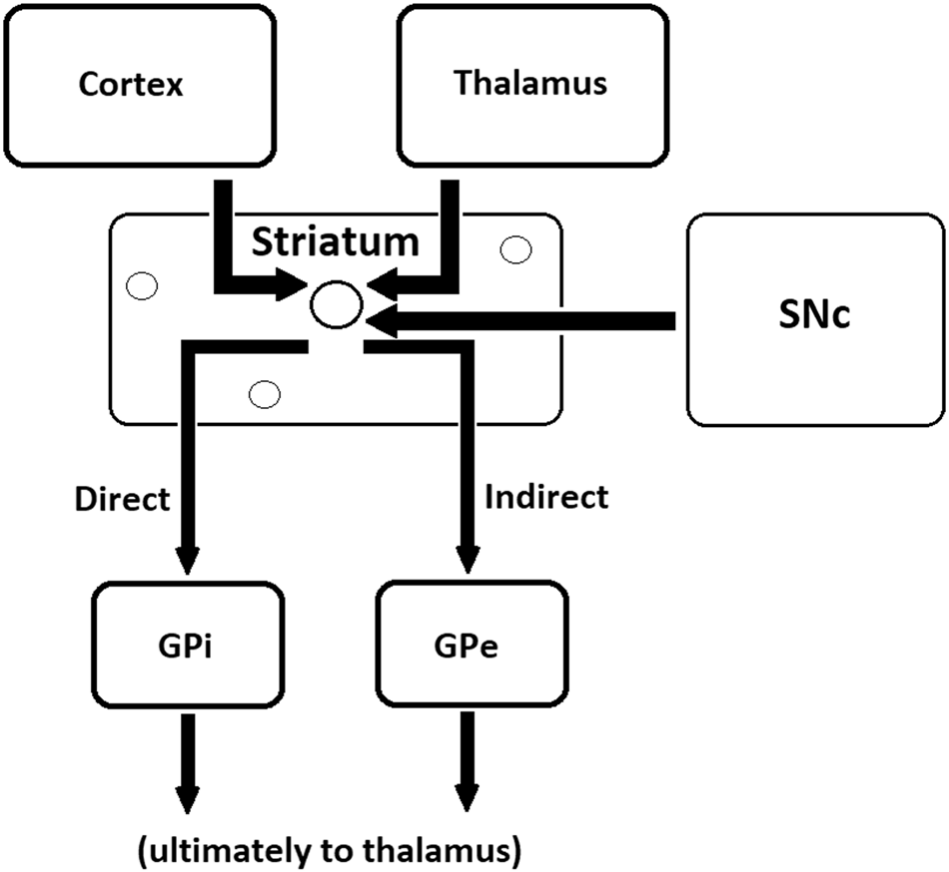
Schematic diagram of the striatum, its various input pathways, and the beginning of the well-characterized direct and indirect output pathways. Striosomes are the site of integration of input from the substantia nigra pars compacta (SNc), thalamus, and cortex, and are prime candidates for the central cognitive pattern generators whose dysfunction may underlie delusions. The matrix, composed primarily of cholinergic interneurons, surrounds the striosomes and modulates input. Output from the striatum is through medium spiny neurons which target the internal globus pallidus (GPi) or the external segment of the globus pallidus (GPe) and eventually, the thalamus^146,147^.

In connection with the dopaminergic hypothesis of psychosis, research on addiction has shown that nicotine drives a much greater increase in phasic signaling by DA in the NAc than in the dorsal striatum which contains the caudate nucleus and putamen^137^; furthermore, it has been shown that there is a reduced density of cholinergic interneurons in the ventral striatum in schizophrenia spectrum disorders^138,139^. The NAc expresses α6 subunit-containing nAChRs, rather than the α4 subunit-containing receptors that constitute the majority of dorsal striatum nAChRs^140^. Notably, a consistent finding has been that of nicotine serving the role of reinforcing reward rather than affecting the dorsal striatum where the putative central cognitive pattern-generating striosomes reside (see Fig. 1). This is consistent with nicotine being addictive but having no effect on positive symptoms and with its documented effect on mesolimbic circuitry and brain regions associated with reinforced behavior, rather than feedback-insensitive behaviors like those produced by central pattern generators. Mesolimbic modulation is also consistent with improvement in the negative symptoms sometimes seen with nicotinic agonism^141^ but not in positive symptoms. α7 nAChR agonists have also been shown to improve both negative symptoms and cognition in schizophrenia spectrum disorders^142^. Notably, it has been shown that cholinergic interneurons in the dorsal striatum are responsible for this modulation^143^. Furthermore, the linkage of α7 nAChRs (widely distributed in cortex) with schizophrenia spectrum disorders has been established for some time by GWAS studies^88^.

**Fig. 2:**
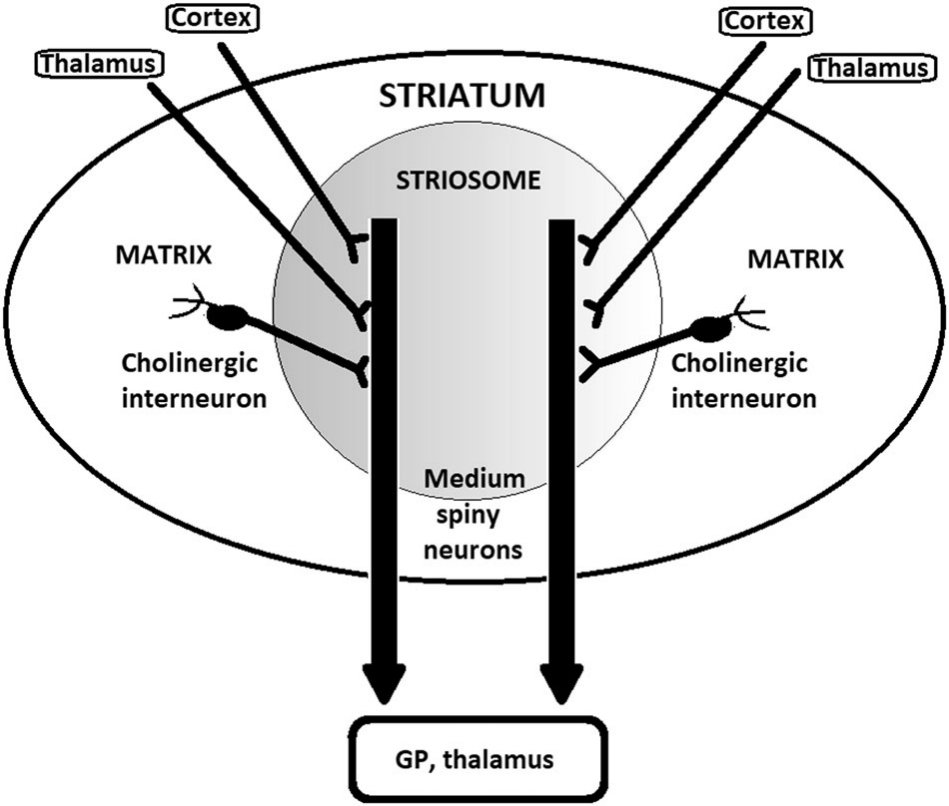
Detail of the striosome. Dopaminergic inputs are received from the SNc and glutamatergic inputs from the cortex and thalamus. The cholinergic system has a key role in the integration of these signals, which are modulated by cholinergic interneurons in the matrix surrounding the striosomes, and output is mediated by medium spiny neurons, which are GABAergic neurons forming the direct and indirect pathways (thick vertical arrows) depicted here and in Fig. 1. See also refs ^146,147,230^.

The model proposing dopaminergic projections from the VTA to the ventral striatum (the mesolimbic circuits) is no longer well-supported, as shown by the meta-analysis of positron emission tomography (PET) data and a review of all available imaging and anatomic data^144,145^. A more likely picture is the central involvement of dysfunction of the nigrostriatal circuits which make synaptic contacts with striosomes in the dorsal striatum. Striosomes are histochemically recognizable striatal compartments within the striatum^146–148^ and will be a key region to further discuss in terms of their putative involvement in delusional thinking (see Figs 1 and 2).

Cholinergic neuronal cells represent only 2% of cells in the striatum but appear to establish synapses on a large fraction of striatal cells^149^. There is anatomical and pharmacologic evidence for this role. By identifying cells in the striatum with choline acetyltransferase (ChAT) immunoreactivity, it was shown that they are most frequently found in the periphery of striosomes where they are ideally positioned to modulate nigrostriatal dopaminergic inputs; there are far fewer in the putamen^150^. Muscarinic M4-agonism alleviates the amphetamine model of psychoses in rodents, with the dorsal striatum as a preferential target^127^. It has been shown that medication-naive patients with schizophrenia spectrum disorders have reduced levels of muscarinic receptors, particularly in the striatum, as to be expected in individuals having difficulty adapting to uncertainty and rapidly updating beliefs^126^.

**Fig. 3:**
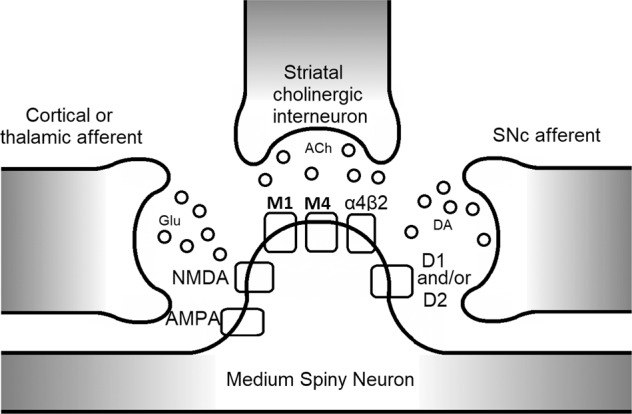
Detail of the most relevant synaptic contacts within the striosome on medium spiny neurons. While it is not a comprehensive diagram of all receptors or all interactions between these neurons, the diagram is intended to highlight the inputs from the cholinergic interneurons, as well as the cortex, thalamus, and substantia nigra pars compacta (SNc), all converging on the medium spiny neuron. Importantly, here is where ACh exerts its effect on prediction error, modulating prediction error by boosting bottom-up signals.

Striosomes are the most likely central pattern generators, e.g. responsible for species-specific stereotypies. If some delusions are indeed cognitive stereotypies arising from hyperfunction of striosomes functioning as cognitive central pattern generators, we can make strong inferences and predictions about the behavior of delusions under various experimental manipulations (see Fig. 3). Stereotypies are well known to be intensified or prolonged by increasing dopaminergic input to, or tone in, the striatum, for example in amphetamine paradigms, both experimentally and in stimulant abuse in humans. Increasing cholinergic transmission in the dorsal striatum stops stereotypies more quickly; decreasing cholinergic transmission by blocking postsynaptic receptors with muscarinic antagonists increases the length of stereotypies^151^ and striatal cholinergic interneurons modulate hyperdopaminergic dyskinesias^152^.

This picture is complicated by the differential function of muscarinic and nicotinic receptors. In the striata of patients with schizophrenia spectrum disorders there are fewer M1-type muscarinic receptors^153^. M1 receptors are not located on cholinergic interneurons and they do not serve an autoreceptor function^154^. However, there are presynaptic non-M1 receptors on cholinergic interneurons^154^. There is also greater nicotine binding in schizophrenic patients, presumably on the postsynaptic target neurons^155^. One would therefore expect that increasing muscarinic or decreasing nicotinic neurotransmission would improve patients suffering from delusions. The striatum is primarily composed of two classes of spiny projection neurons (SPNs): the striatonigral and striatopallidal SPNs, which express D1R and D2R, respectively. Within striosomes there are more D1R-expressing than D2R-expressing SPNs, whereas in the matrix there are more D2R-expressing than D1R-expressing SPNs (Figs 2 and 3).

## ACh in delusional illness: PD

Psychosis is a non-motor and late complication of PD. It predicts Lewy body pathology in the brain cortex^156^ and shows a variable response to antipsychotic agents. In fact, psychosis in PD presents with marked heterogeneity suggesting the involvement of multiple neurotransmitter systems in its genesis. Recently, Factor and coworkers were able to delineate multiple subtypes of PD psychosis^157^. Delusions are present in about 6–16% of patients, with Othello syndrome, grandiose, religious and guilt types being the most frequent forms of delusion^157^.

The exact mechanism of delusional symptoms in PD patients is still obscure and is not explained by current ideas on the development of psychosis in these patients^158^. However, the role of the cholinergic system in PD is made clear by the finding of a strong, statistically significant correlation between hallucinations (but not delusions) and freezing of gait^159^, both coexisting with cognitive decline. Cholinergic mechanisms are involved in the development of hallucinations^160^ but, interestingly, delusions are not associated with cognitive decline^161^ and some patients present with pure delusional syndromes in the absence of perceptual disturbances^162,163^. The development of delusions does not appear to have a cholinergic basis, is related to dopaminergic medications, and is reversible upon discontinuation of the offending drug^164^.

## ACh in other neuropsychiatric illnesses

It should be noted that although the pathophysiology of delirium is thought to rely on a disturbance in cholinergic neurotransmission^165^, given the acuity and rapid course of the illness and medical fragility of those suffering from it, there is a dearth of imaging studies directly examining this question and certainly in looking at the specific pathogenesis of delusions in this condition^166,167^. It is intriguing that there is a case report of a patient with delirium secondary to a urinary tract infection developing a Capgras delusion, presumably as part of delirium, which resolved in days with the cure of the organic illness^168^.

## The neuronal cell and circuitry levels

### Huntington disease

The occurrence of neuropsychiatric symptoms in HD, including positive symptoms of psychosis, is common^169^ and generally under-appreciated. While almost all HD patients develop neuropsychiatric symptoms during their illness, a recent survey found that psychosis is the presenting feature in 11% of cases and that delusions occurred in 11.5% of patients^170^. Obsessive compulsive symptoms are more common than delusions^171^, consistent with the theory of striosomal hyperfunction as underlying both cognitive stereotypies (obsessions, delusions) and motor behaviors, as in obsessive compulsive and tic disorders. It is also curious that, similar to patients with psychosis, HD patients often have poor insight into their deficits, both cognitive and motor^172^.

The etiogenesis of HD is well characterized^173^, with the expansion of a trinucleotide repeat in the Huntington gene, causing a toxic gene product to accumulate in and cause selective death of the GABAergic medium spiny neurons, making psychotic symptoms difficult to explain in terms of the dopaminergic model of psychosis. These neurons constitute the majority of cells in the caudate^174^. As a result, as the disease progresses the classic finding is radiologically visible atrophy of the caudate heads, although post-mortem studies typically show degeneration in many brain regions^175–178^. While in terms of cell count, cholinergic interneurons are spared, ChAT levels drop, suggesting dysfunction of these cells^179^. Consistent with the current model of the computational function of striatal cholinergic interneurons, with decreasing activity of these cells we should expect an intensification of stereotypies and rigid beliefs. Therefore, a test of the cholinergic neurotransmission aspect of the striosomal hypothesis of delusions would be to investigate the association between the extent or distribution of ChAT loss and the onset or intensity of psychiatric symptoms including delusions; to the best of our knowledge no such data is available as yet. Similarly, delirium and many dementing illnesses are canonically considered to be disruptions in ACh signaling. To our knowledge, there has been no attempt to link ACh signaling directly in the basal nuclei, and the caudate in particular, to the severity of the pathology in these illnesses, including delusions. Given the increasing importance of this nucleus in neuropsychiatric illness and the argument here for its role in delusion, this aspect should be further explored.

### ACh and prediction error

A great deal of what we know about cognitive approaches to understanding delusions has been pioneered by the work of Corlett^12–16^. Brains are inference engines which construct hypotheses to predict or interpret stimuli (“top-down” processing), and when there is a discrepancy between prediction and perception, generate an error message (a prediction error signal, “bottom-up” processing)^180,181^, see review in ref. ^182^. This Bayesian (probabilistic) inference mechanism is understood to be defective in various forms of psychoses, particularly in schizophrenia spectrum disorders, either because of abnormal signaling of the prediction errors, abnormal sensory inputs, or both, ultimately resulting in dysfunctional predictive coding. Applying this concept to the empirical dysfunction observed in delusional illnesses may show where this computation is instantiated and ultimately suggest corrective measures that could be used in therapeutic approaches.

Thus far, research on the prediction error signal has largely focused on glutamatergic or dopaminergic neurotransmission. Predictive coding has been associated with gain of control of postsynaptic glutamatergic excitatory transmission mediated by NMDA receptors and dopaminergic neuromodulation^180^. However, as schematically depicted in Fig. 3, ACh has an important role in modulating this signal; the behavior of VTA circuitry is explained by a computational model showing that the endogenous neurotransmitter ACh and the exogenous full nicotinic agonist nicotine both effectively desensitize nAChRs and cause increased reward signals, i.e. decreasing ability to predict reward^183^. These effects are mediated by α4β2 nAChRs on GABAergic neurons acting on dopaminergic neurons—also containing α4β2 nAChRs on their surface—in the VTA (reviewed in ref. ^183^). This has been demonstrated in an experimental paradigm using galantamine application in combination with EEG measurements, by showing that ACh boosts bottom-up prediction errors, which are preferentially observed under conditions of increased uncertainty^184,185^. In this way the brain’s purported hierarchical Bayesian prediction function can be tuned to increase input from top-down or bottom-up data, for optimal predictions under varying conditions^186^. It is intriguing that one of the hallmarks of delusional ideation is pathologic conviction (certainty when it is not justified), especially as it relates to the striosomal hypothesis of delusions. Yamanaka and coworkers argue that thalamostriatal neurons project to striatal cholinergic interneurons, and that this system generates an “associability” signal, the certainty of which is modulated by cholinergic neurotransmission^187^. It is known that dopamine release from these thalamocortical projections is tonic and suppresses firing of striatal cholinergic interneurons, but the significance of this in the context of illness is unclear^188^.

It is not clear whether ACh can be accurately measured by magnetic resonance spectroscopy (MRS) in awake subjects. MRS allows the measurement of molecules in the brain by detecting the specific magnetic fields of protons as they are shifted by moieties in those molecules. While two neurotransmitters (glutamate and GABA) can be accurately and reliably measured, neurochemical and technical difficulties have so far made this challenging for ACh. First, it is the choline moiety (not the full ACh molecule) that is detected, and a substantial fraction (likely the majority) of this signal can be assumed to come from free choline being used for cell membrane biosynthesis. Second, there is less ACh present than the two other neurotransmitters mentioned, and this worsens the signal to noise ratio. Nonetheless, in rat, MRS-measured choline was shown to correlate with directly (chemically) measured ACh^189^. However, functional changes may not be measurable, as task-driven changes in MRS-measured choline were similar to the presumed unrelated metabolite N-acetyl-aspartate, suggesting that dynamic MRS measurements may still not be sufficiently sensitive and/or reliable^190^.

Linking up with the framework of Bayesian reasoning and prediction error, it has been argued that ACh modulates prediction error signals and tunes the brain’s updating in response to varying degrees of uncertainty. That is, in predictable environments, ACh enhances bottom-up signaling (to enhance the salience of sense data) and in unpredictable environments, it dampens it. This model is experimentally supported by increased sensory cortex response in an oddball paradigm with human subjects taking galantamine^184^. Galantamine also increases the rate of updating in a spatial beliefs model (using an eye-tracking paradigm)^185^. Consequently, a hypocholinergic state would be expected to slow belief updating (as in delusional belief) and an increase in cholinergic tone would improve it, as shown with galantamine^191^. Acetylcholinesterase inhibitors (which increase ACh neurotransmission) have been used off-label to improve cognition in schizophrenia^192–195^. The obvious inference is that the decreased cholinergic neurotransmission, consistent with what is seen in α7 nAChR loss-of-function mutations in animal models, would result in decreased ability to adapt to environments based on predictability and consistent low weight placed on unexpected data in predictable environments, relative to top-down beliefs. Cognitively, this could be envisioned as someone who is convinced of the predictability of the world despite sense data inconsistent with this belief.

An additional role of ACh is worth mentioning. In a seminal paper, Moran and coworkers showed that ACh is a neuromodulatory transmitter involved in perception and learning under uncertainty^184^. Using computational simulations and electroencephalography the authors suggested that ACh enhances the precision of bottom-up synaptic transmission, optimizing the gain of pyramidal cells^184,185^.

## A unifying hypothesis: delusions are in part caused by hyperdopaminergia and overactive striosomes in the dorsal striatum, modulated by cholinergic interneurons

One shortcoming of delusion theories thus far is the minimal effort taken to explain the specificity of polythematic content, with delusion being clustered into predictable themes across cultures. The dopamine theory of psychosis is well-established; dopamine blockade improves positive symptoms and dopamine agonists induce or worsen positive symptoms. What remains under-appreciated is the anatomy of dopamine modulation in psychosis, with recent evidence pointing toward the nigrostriatal dopaminergic pathway and focusing on the dorsal striatum as relevant in the production of psychotic symptoms^145^. Specific models of delusional belief show that changes in activity in the dorsal striatum as observed with fMRI correlate with the amount of delusional ideation in illness^196^.

One argument for the source of delusional themes advances striosomes as the explanation for the content of delusions^148^, conserved as they are cross-culturally, strongly suggesting a biological basis. As illustrated in Figs. 1–3, striosomes are clusters of specialized neurons within the caudate which receive dopaminergic projections from the substantia nigra pars compacta^148^. Striosomes are known to be responsible for specific motor programs (stereotypies) which have clear evolutionary importance and are not subject to modification and are highly reward-insensitive. For example, individual striosomes in rodents have been isolated and shown to be responsible for specific stereotypies, e.g. parts of the grooming chain^197^. The basal ganglia are evolutionarily older than the cortex and off-loading repetitive, evolutionarily important actions makes economic sense for the brain. Dopamine agonism has been shown to cause growth in the striosomes, the degree of which in turn is correlated with the increase in stereotypy frequency^198,199^.

While thus far striosomes have only been shown to be responsible for motor programs in non-human animals, it is speculated that in humans they may also be responsible not only for stereotypies, but also for cognitions which are of similar evolutionary importance and reward-insensitive. The fit with delusional themes is immediately apparent—for instance, identification of conspiring competitors, possible mates, or cheating on one’s own mate. Furthermore, delusions are responsive to dopamine blockade or agonism, and in other illnesses like OCD or Tourette syndrome, there is a clear crossover between repetitive motor and cognitive patterns^200^. Some of the themes of OCD overlap with delusions and indeed sometimes there is damaged, but not absent, insight, making diagnosis difficult and resulting in discussion at one time about adding a schizo-obsessive diagnostic category to DSM^201,202^. Tourette is a related illness with a specific reward-insensitive behavior that often responds to antipsychotics. In cases where it does not, the nicotinic antagonist mecamylamine has been found to sometimes be effective^203^. The argument is strengthened by our increasingly detailed knowledge of the complex architecture of the dorsal striatum, with considerable evidence that matrix and striosome respond differently to the same inputs^147,204^. There are differences depending on nicotinic receptor subtype within the striosomes, and differential activation may be what drives fixed motor pattern (stereotypies)^205^. The role in aberrant movement of dopaminergic hyperstimulation of the dorsal striatum and its modulation by cholinergic inputs has been well-established^152^. In fact in mice with amphetamine-induced stereotypies and poor response to environmental cues, direct electrophysiology shows that cholinergic modulation of striosomes is disrupted, again suggesting that ACh in the dorsal striatum serves to increase sensitivity to bottom-up information and thus predispose to belief updating. This has an obvious parallel in amphetamine-induced psychosis in humans, as humans develop tics and stereotypical behaviors in response to intoxication with dopamine agonists^206^. If as has previously been argued^207^ we can extend the animal models of striosome-originated stereotypies to delusions, taking stereotypies as rigid behaviors, a kind of “motor delusion” (or considering delusions as “cognitive stereotypies”), then this would explain the neuroanatomy underlying delusions in psychosis and dopamine agonist intoxication. The striosomal model therefore argues that the specific themes of delusions (specificity), and their insensitivity to new information (conviction) is the result of hyperdopaminergic, over-driven striosomes. It should also be noted that computational studies of cognition have noted attractor basin-like dynamics demonstrated by delusion formation^208^, and striosomes are an excellent candidate for the physical instantiation of this phenomenon.

In terms of neurotransmission, it is suggestive that the highest concentration of ACh in the brain is in fact in the dorsal striatum^209^, and given the role of this neurotransmitter in modulating striatal prediction error, one would expect cholinergic antagonism to worsen delusions, which in fact is what is observed, particularly in delirium and AD dementia, as well as in some cases of Huntington and iatrogenic psychoses. Thus, the striosomal theory of delusional content accounts both for the dopamine theory of psychosis, and deviations from the model where the disturbance is cholinergic.

It should be noted that the dorsal striatum accounts for only part of the hypothesis; namely, the specificity of polythematic delusions. The poor insight present in delusions, as well as somatic and misidentification delusions, very likely result from functional lesions of the right frontal lobe. Indeed, that somatic or misidentification delusions (e.g. Capgras syndrome) result from lesions of the right frontal lobe is one of the best supported observations about the anatomy of delusions so far^210,211^. Given these putative roles, it may be the case that a right frontal lesion without striosomal hyperfunction results in somatic delusions, whereas striosomal hyperactivity in the dorsal striatum occurring without right frontal deficits would induce thematically specific cognitive or motor behaviors, with preserved insight, conditions that are worsened by dopamine agonists and improved by dopamine antagonists. This is a description of OCD and Tourette syndrome, which we know involve disruptions to striatal signaling^212^ and often respond clinically to dopamine blockade.

It is important to note that the cognitive approaches for understanding delusional thinking using a two-factor hypothesis, mainly proposed by Corlett^12–16^, have been recently revisited by this author^213^.

### New directions for investigating and treating delusions

Over the past two decades there has been a focus on developing cholinergic (especially nicotinic) modulators for schizophrenia spectrum disorders and AD that have yielded inconsistent results^214^, as well as case reports with acetylcholinesterase inhibitors^193,215^. It may be that the best role for such molecules is as an adjunct, given the cholinergic system’s role in modulating uncertainty and updating, while dopamine is more central to the etiology of psychosis. Recent pharmacological evidence has provided new insights into our understanding of cholinergic signaling in brain and have put the α4β2 and the α7 nAChRs in a center-stage position as possible targets for therapeutic intervention in neuropsychiatric diseases with cognitive deficits including autism spectrum disorders, nicotine addiction, schizophrenia spectrum disorders^88^, AD, and PD^56,216–218^. For instance, rivastigmine, a drug that increases cholinergic tone by inhibiting the enzyme cholinesterase, is effective for dementia, whereas the use of Donepezil is still in the realm of investigation^218^. Activation of the α7 nAChRs by selective ligands enhances cognition in schizophrenic and AD patients^74,84,85,219^.

Another set of compounds gaining momentum is the wide spectrum of allosteric modulators, new leads as therapeutic agents for the treatment of neuropsychiatric diseases with cognitive deficits^220^. These ligands exert their action in the presence of the endogenous agonist, and by definition do not target the canonical (“orthosteric”) nAChR agonist or competitive antagonist recognition sites on the receptor molecule, but bind to distant, non-orthosteric, allosteric sites which nonetheless affect the response of the orthosteric site. There are essentially two types of allosteric modulators, most widely studied for the α7 nAChR, which either potentiate (positive allosteric modulators, “PAMs”) or inhibit (negative allosteric modulators, or “NAMs”) the response of agonist binding to the orthosteric site^221–223^. A third type of compounds corresponds to the “silent” allosteric modulators (“SAMs”), which inhibit the PAM responses without affecting the orthosteric responses. Some authors include a fourth category, namely the allosteric agonists, but clearly these should not be classified as modulators: they are agonists which bind not to the orthosteric but to the allosteric site. Presumably, endogenous allosteric modulators occur in brain. In animal studies, PAMs acting on α7 nAChRs, like PNU-120596 and galantamine, have been shown to enhance performance in various cognitive and recognition memory tasks^224^. The prevalent effect of PAM compounds is to increase the probability of the nAChR channel open state, thereby increasing its lifetime and the number of open channel events^225^. Furthermore, the PAM compounds CCMI, PNU120596, and A582941 reverse the sensorimotor gating impairment evoked by MK-801 based on the pre-pulse inhibition of the startle response, thus showing a beneficial effect on sensorimotor gating and some aspects of cognition^226^.

There is also a great wealth of data on clinical and preclinical observations where muscarinic receptors are targeted. The most conspicuous of these muscarinic agents is xanomeline^227^ and orthosteric muscarinic ACh receptor agonist which binds to M1 and M4 receptors. It has proven efficacy in reducing psychotic symptoms in schizophrenia^228^ and AD^229^, including an effect on delusional thinking. Recently, xamomeline has been used in conjunction with tropsium, a peripheral muscarinic antagonist in hospitalized patients with schizophrenia that experience an exacerbation of symptoms. This was a randomized, double-blinded, placebo-controlled Phase-2 trial (clinicaltrials.gov, NCT03697252).
